# Hypertension as predictive factor for bevacizumab-containing first-line therapy in metastatic breast and colorectal cancer in BRECOL (GEICAM/2011-04) study

**DOI:** 10.1007/s12094-024-03411-w

**Published:** 2024-04-05

**Authors:** Álvaro Rodríguez-Lescure, Javier Gallego, Pilar Garcia-Alfonso, Bartomeu Massuti, Raúl Márquez, Lourdes Calvo, Pedro Sánchez-Rovira, Antonio Antón, José Ignacio Chacón, Eva Ciruelos, Jose Juan Ponce, Ana Santaballa, Manuel Valladares-Ayerbes, María Rosario Dueñas, Vicente Alonso, Jorge Aparicio, Sara Encinas, Luis Robles, María José Escudero, Rosalía Caballero, Susana Bezares, María Victoria García-Ortiz, Teresa Morales-Ruiz, Juan de la Haba-Rodriguez

**Affiliations:** 1 Medical Oncology Department, Hospital General Universitario de Elche, Carrer Almazara, 11, 03203 Elche, Alicante Spain; 2 GEICAM Spanish Breast Cancer Group, Madrid, Spain; 3 Instituto de Investigación Sanitaria Gregorio Marañón, Facultad de Medicina de la Universidad Complutense de Madrid, Hospital General Universitario Gregorio Marañón, Madrid, Spain; 4 Instituto de Investigación Sanitaria y Biomédica de Alicante (ISABIAL), Hospital General Universitario Dr. Balmis, Alicante, Spain; 5 MD Anderson Cancer Center Madrid, Madrid, Spain; 6 Complejo Hospitalario Universitario de A Coruña, A Coruña, Spain; 7 Hospital Universitario de Jaén, Jaén, Spain; 8 Instituto de Investigación Sanitaria de Aragón (IISA), Hospital Universitario Miguel Servet, Universidad de Zaragoza, Saragossa, Spain; 9 Hospital Universitario de Toledo, Toledo, Spain; 10 Hospital Universitario, 12 de Octubre, Madrid, Spain; 11 Hospital Universitario y Politécnico La Fe, Valencia, Spain; 12 Instituto de Biomedicina de Sevilla (IBiS), Hospital Universitario Virgen del Rocío, Seville, Spain; 13 Instituto Maimónides de Investigación Biomédica (IMIBIC), Hospital Universitario Reina Sofía, Universidad de Córdoba, Córdoba, Spain; 14 Oncology Biomedical Research National Network (CIBERONC-ISCIII), Madrid, Spain; 15 Maimónides Biomedical Research Institute of Córdoba (IMIBIC), Córdoba, Spain; 16 Medical Oncology Department, Reina Sofía University Hospital, Córdoba, Spain; 17 Cancer Network Biomedical Research Center (CIBERONC), Madrid, Spain; 18 Department of Genetics, University of Córdoba, Córdoba, Spain; 19 Reina Sofía University Hospital, Córdoba, Spain

**Keywords:** Metastatic breast cancer, Metastatic colorectal cancer, Bevacizumab, Blood pressure

## Abstract

**Background:**

Retrospective data suggest an association between bevacizumab efficacy and the incidence of arterial hypertension (AHT). Additionally, epigenetic mechanisms have been related to AHT.

**Methods:**

This prospective observational study conducted by GEICAM Spanish Breast Cancer Research Group included metastatic breast (MBC) or colorectal (mCRC) cancer patients treated with bevacizumab-containing chemotherapy as first-line treatment. Blood pressure (BP) levels were measured (conventional and 24-h Holter monitoring) at baseline and up to cycle 3. Primary endpoint assessed BP levels increase as predictive factor for progression-free survival (PFS). Germline DNA methylation profile was explored in pre-treatment blood samples; principal component analysis was used to define an epigenetic predictive score for increased BP levels.

**Results:**

From Oct-2012 to Jul-2016, 143 (78 MBC and 65 mCRC) patients were included. The incidence of AHT according to guidelines was neither predictive of PFS nor of best overall tumor response (BOR). No statistically significant association was observed with systolic BP nor diastolic BP increment for PFS or BOR. Grade 3 and 4 adverse events were observed in 37 and 5% of patients, respectively. We identified 27 sites which baseline methylation status was significantly associated to BP levels increase secondary to bevacizumab-containing chemotherapy.

**Conclusions:**

Neither the frequency of AHT nor the increase of BP levels were predictive of efficacy in MBC and mCRC patients treated with bevacizumab-containing chemotherapy.

**Clinical trial registry:**

ClinicalTrials.gov Identifier: NCT01733628.

**Supplementary Information:**

The online version contains supplementary material available at 10.1007/s12094-024-03411-w.

## Introduction

Arterial hypertension (AHT) is a common toxicity with bevacizumab (BVZ)-containing chemotherapy (CT/BVZ), and is usually easily managed with common medical treatment. Previous meta-analysis showed that severe AHT requiring medical intervention, was noted in 11–16% of cancer patients treated with BVZ [1].

Several mechanisms have been postulated for AHT secondary to BVZ, such as vascular endothelial growth factor (VEGF) acting as blood pressure (BP) homeostatic factor, and VEGF signal antagonism correlating with inhibition of nitric synthase [2]. The AHT incidence with BVZ and the underlying molecular mechanisms led to propose that BP levels elevation could act as a biomarker for efficacy of VEGF signal inhibition [3].

AHT is usually graded according to the National Cancer Institute Common Terminology Criteria for Adverse Events (NCI-CTCAE), mostly based on therapeutic interventions rather than BP levels; consequently, AHT may be underestimated when graded toxicity is the source. On the other hand, AHT on BVZ therapy is mainly detected with treatment administration or symptoms appearance, and BP levels elevation upon receiving BVZ is frequently observed within the first cycle [4]. Hence, non-symptomatic AHT or non-clinically significant increased BP levels may be underreported in clinical trials and underregistered by clinicians. In fact, most of the studies to confirm that AHT correlates to BVZ efficacy have a retrospective design. Given the lack of standardized follow-up of BP levels in the medical oncology community and the above-mentioned methodological biases, the true incidence of BVZ-induced AHT and its potential relationship with outcomes remain unclear.

The antitumor activity of BVZ is especially related to the VEGFR2-mediated angiogenesis inhibition [5]. One of the biological models that most closely resembles the anti-VEGF/VEGF receptor (VEGFR) action of antiangiogenic therapies is preeclampsia [6]. Among the epigenetic mechanisms potentially related to variations in susceptibility to gestational AHT is DNA methylation [7]. Recent studies have identified several genes whose hypomethylation is associated with early onset of preeclampsia.

The aim of our study was to prospectively assess the increase in BP levels, measured by conventional methods and 24-h Holter monitoring, as a predictive factor for CT/BVZ in first-line therapy of metastatic breast (MBC) and colorectal cancer (mCRC) patients in terms of progression-free survival (PFS). We also looked for predictive methylation markers of AHT secondary to antiangiogenic treatment.

## Materials and methods

### Study design

We conducted a multicenter, prospective, observational study to evaluate in real-life AHT incidence as a predictive factor for PFS in patients treated with CT/BVZ as first-line therapy.

The incidence of BP levels increase as a predictive factor of tumor response, was a secondary objective. Exploratory objectives included the AHT secondary to BVZ-related biomarkers in pre-treatment blood samples.

The study was conducted in compliance with the ICH-GCP guidelines and the Declaration of Helsinki. The study was reviewed and approved by the sites independents ethics committees and Health Authority in Spain. Written informed consent was obtained before any study-related procedures. ClinicalTrials.gov identifier: NCT01733628.

### Eligibility criteria

Eligible patients met the following criteria: women and men, ≥18 years, diagnosed with MBC or mCRC and indication of CT/BVZ as first-line therapy, ECOG (Eastern Cooperative Oncology Group) <2 and, measurable disease according to RECIST (Response Evaluation Criteria In Solid Tumors) version 1.1. Patients had to be treated with BVZ every 2–3 weeks plus fluoropyrimidines and either oxaliplatin or irinotecan if mCRC or plus paclitaxel or capecitabine if MBC. The treatment decisions were taken according to routine clinical practice.

Patients were excluded if they received any previous systemic CT for advanced disease, BVZ administration, treatment with an investigational medication within 30 days prior to study inclusion; life expectancy <3 months (mo.); pregnancy or breastfeeding; or abnormal relevant organ functions.

### Study procedures

Baseline assessments were performed within 14 days before CT/BVZ started and included physical examination, vital signs, ECOG and blood analyses as per routine clinical practice, and pregnancy test. Data related to concomitant medication and cardiovascular risk factors were recorded.

Measurable disease was evaluated per routine clinical practice (≤35 days before CT/BVZ started, and every 9–12 weeks during treatment period). Follow-up visits were performed from the fourth cycle on, with tumor assessments until either progressive disease (PD), a new anticancer therapy without BVZ started, or death from any cause.

BP was measured within 2 weeks before starting CT/BZ, and the first day (D1) of the first three cycles (C1–C3), through 24-h Holter monitoring (starting 2 h before treatment administration). Additionally, BP was measured through conventional method three times per week on C1–C3; the first weekly measurement was performed at hospital before treatment administration (D1 of the cycle). As a control method, patients recorded in a diary the values (systolic [SBP] and diastolic [DBP] BP levels and pulse rate) obtained at home, a pharmacy or primary healthcare center. BP was measured following the European Society of Cardiology and Hypertension Guideline 2007 [8], and values for AHT diagnosis are included in *Supplementary Table 1*. A period of 3–4 weeks of CT/BVZ was considered as one cycle to obtain uniformity in data because of the different CT/BVZ regimens.

The adverse events (AEs) of grade 3 (G3) and 4 (G4) related to treatment were recorded during C1–C3 and reported considering the NCI-CTCAE [9] version 4.0. All serious AEs (SAEs) were also collected.

The treatment was continued until PD, unacceptable toxicity, patient’s decision, pregnancy, or any other reason that prevented patient’s treatment according to investigator’s judgment, whatever occurred first.

Germline DNA methylation (gDNAmeth) profile was explored in pre-treatment peripheral blood samples, in a central laboratory (*Supplementary Methods*).

### Statistical considerations

It was estimated that developing AHT related to CT/BVZ would decrease the risk of PD by 50% and that 20% of patients would develop AHT when treated with CT/BVZ. With an inclusion time of 12 mo., a follow-up period of 24 mo., alpha error of 5%, power of 80% and a drop-out rate of 10%, 137 patients were needed.

A descriptive analysis was performed for demographic data and clinicopathological characteristics. No imputation of missing data was made. All the hypotheses’ tests performed were bilateral and with a significance level of 0.05. The Statistical Analysis System (SAS) software was used.

The final analysis was performed considering the BP levels measurement through 24-h Holter monitoring and records in patient’s diaries for correlation with PFS and tumor response. To evaluate the increase in BP levels, the differences in absolute value between its measurements at C1–C3 and the baseline assessment were analyzed considering several cut-off points (≥5, ≥10, ≥15 and ≥20 mmHg), the SBP and DBP levels, and the difference between both. A cut-point model was performed to select the optimum cut-off points, and the statistical significance was calculated with a permutation test, repeating the cut-off points selection process with randomly permuted response.

The Kaplan–Meier (K–M) method and log-rank test were used to compare survival curves. A multivariate Cox regression analysis was performed, including in the model the difference in SBP (24-h Holter) ≥10 mmHg (yes/no) and clinically relevant variables.

An interim analysis was performed in Dec–2013, with 43 evaluable patients who had completed C1–C3 (25 MBC and 18 mCRC). Its purpose was to evaluate the distribution of patients by tumor type and the actual AHT incidence, and no further actions were needed after the evaluation of results.

Linear model fitting and differential methylation analysis was performed using the eBayes moderated t-statistic (for outcome variable) by LIMMA (“Linear Models for Microarray Analysis”) package [9] for the R statistical software. Raw *P*-values were adjusted using the Benjamini-Hochberg’s procedure, and a False Discovery Rate (FDR) cut-off of 0.05 and deltaBeta ≥|10|% in the outcome-related analyses was used as statistically significant threshold. Principal Component (PC) analysis was used for defining a methylation score predictive of elevated BP levels.

## Results

### Patients’ demographics and baseline disease characteristics

**Fig. 1:**
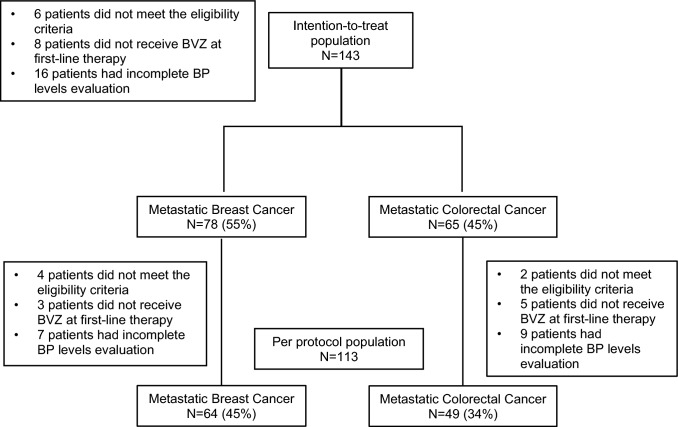
Consort diagram

Between Oct-2012 and Jul-2016, 143 patients were included across 11 sites in 8 Spanish regions, 78 (55%) patients with MBC and 65 (45%) with mCRC. Baseline patients’ characteristics are detailed in Table 1. Cardiovascular risk factors included AHT 56 (39%), dyslipidemia 40 (28%), glucose metabolism alteration 11 (8%), and obesity 4 (3%), were the most frequent comorbidities in the *ITT* population. One hundred thirty-five patients were included in the *safety* population as they received at least one dose of CT/BVZ and the *per protocol (PP)* population included 113 patients. The patients’ distribution within the study populations is detailed in Fig. 1*.* The reasons for ending CT/BVZ are shown in *Supplementary Table 2*.

**Table 1:** Patient’s demographics and baseline disease characteristics

	MBC (*n* = 78)	mCRC (*n* = 65)	Total (*n* = 143)
	*n* (%)	*n* (%)	*n* (%)
Gender			
• Female	77 (99)	25 (38)	102 (71)
• Male	1 (1)	40 (62)	41 (29)
Age, years (median; range)	56 (32–82)	67 (41–85)	61 (32–85)
ECOG Performance Status			
• 0	48 (62)	19 (29)	67 (47)
• 1	29 (37)	43 (66)	72 (50)
• Unknown	1 (1)	3 (5)	4 (3)
Menopausal status at study entry			
• Premenopausal	24 (31)	3 (12)	27 (26)
• Postmenopausal	53 (69)	22 (88)	75 (74)
Weight, Kg (median; range)	64 (43–110)	74 (49–114)	68 (43–114)
SBP, mmHg (median; range)*	124 (80–180)	130 (92–216)	126 (80–216)
DBP, mmHg (median; range)	75 (50–105)	73 (51–121)	74 (50–121)
• Missing (*n*)	4	5	9
Pulse rate, bpm (median; range)*	79 (61–128)	75 (52–105)	76 (52–128)
• Missing (*n*)	4	8	12
De novo metastatic disease			
• No	61 (78)	31 (48)	92 (64)
• Yes	17 (22)	34 (52)	51 (36)
Biomarker analyses in primary tumor			
• ER+ and/or PgR+ and HER2−	53 (68)	–	–
• ER+ and/or PgR+ and HER2+	2 (3)	–	–
• ER− and PgR− and HER2+	1 (1)	–	–
• ER− and PgR− and HER2−	18 (23)	–	–
• K-RAS mutated	–	35 (54)	–
• K-RAS wild-type	–	23 (35)	–
• Missing	4 (5)	7 (11)	–
Type of metastases			
• Visceral	62 (79)	62 (95)	124 (87)
• Non-visceral	16 (21)	2 (3)	18 (13)
• Unknown	–	1 (2)	1 (1)
Number of metastatic sites			
• 1	15 (19)	22 (34)	37 (26)
• 2	33 (42)	25 (38)	58 (41)
• 3	18 (23)	11 (17)	29 (20)
• ≥4	12 (16)	6 (9)	18 (13)
• Unknown	–	1 (2)	1 (1)
Location of metastatic sites ♣			
• Bone	48 (62)	4 (6)	52 (36)
• Lymph nodes	41 (53)	11 (17)	52 (36)
• Lung/pleura	30 (38)	36 (55)	66 (46)
• Liver	28 (36)	43 (66)	71 (50)
• Skin/soft tissue	6 (8)	–	6 (4)
• Breast or colon/rectum	13 (17)	10 (15)	23 (16)
• Brain	3 (4)	–	3 (2)
• Peritoneum	2 (3)	13 (20)	15 (10)
• Other	6 (8)	10 (15)	16 (11)
Prior therapy for early disease			
• Neoadjuvant and/or adjuvant	58 (74)	23 (35)	81 (57)
• Not applicable	20 (26)	42 (65)	62 (43)
CT regimens in combination with BVZ (*per protocol* population)	*n* = 64	*n* = 49	
• Paclitaxel	43 (67)	–	–
• Capecitabine	19 (29)	1 (2)	–
• Paclitaxel/Carboplatin	1 (2)	–	–
• Gemcitabine/Paclitaxel	1 (2)	–	–
• FOLFOX	–	27 (55)	–
• CAPOX	–	12 (24)	–
• FOLFIRI	–	9 (19)	–
• *Not evaluable*^*⁑*^	*14*	*16*	–
Prior hypertension	*n* = 64	*n* = 49	*n* = 113
• Yes^⁋^	18 (28)	25 (51)	43 (38)
○ G1	3 (5)	9 (18)	12 (11)
○ G2	15 (23)	13 (27)	28 (25)
○ G3	–	1 (2)	1 (1)
○ Grade unknown	–	2 (4)	2 (2)
• No	46 (72)	24 (49)	70 (62)

*MBC* metastatic breast cancer, *mCRC* metastatic colorectal cancer, *ECOG* Eastern Cooperative Oncology Group, *Kg* Kilogram, *mmHg* millimeter of Mercury, *SBP* systolic blood pressure, *DBP* diastolic blood pressure, *bpm* beats per minute, *ER* estrogen receptor, *PgR* progesterone receptor, *HER2* human epidermal growth factor receptor 2, *CT* chemotherapy, *BVZ* bevacizumab, *FOLFOX* 5-Fluorouracil, folic acid and oxaliplatin, *CAPOX* capecitabine and oxaliplatin, *FOLFIRI* 5-Fluorouracil, folic acid and irinotecan, *5FU* 5-Fluorouracil

^*^Measured at medical office as part of baseline physical examination. ^♣^Each patient may have more than one metastatic site. ^⁑^These patients were excluded from the analyses considering the protocol population. ^⁋^Grade of hypertension was classified according to NCI-CTCAE version 4.0

### Treatment exposure

CT regimens in combination with BVZ are shown in Table 1*, a*nd dose modifications in *Supplementary Table 3*.

### Efficacy

With a median follow-up of 8 mo. (range 0.1–47), the median PFS in the *PP* population (*n* = 113) was 9 mo. (95%CI 7–10), 8 mo. (95%CI 7–11), and 9 mo. (95%CI 5–11) in the full population, MBC and mCRC patients, respectively. The most frequent PFS events were PD (62%), another anticancer therapy without BVZ started (23%) and, less commonly, death (4%) (cause unknown in two patients, liver progression in one patient and pulmonary embolism in another patient). The best overall response (BOR) according to RECIST 1.1 is shown in Table 2.

**Table 2:** Best overall response according to RECIST version 1.1 by tumor type

	MBC (*n* = 64)	mCRC (*n* = 49)	Total (*n* = 113)
	*n* (%)	*n* (%)	*n* (%)
Complete response	8 (12.5)	1 (2.0)	9 (8.0)
Partial response	27 (42.2)	26 (53.1)	53 (46.9)
Stable disease	16 (25.0)	10 (20.4)	26 (23.0)
Progressive disease	6 (9.4)	4 (8.2)	10 (8.8)
Unable to be assessed*	1 (1.6)	2 (4.1)	3 (2.7)
Not applicable**	6 (9.4)	6 (12.2)	12 (10.6)

*MBC* metastatic breast cancer, *mCRC* metastatic colorectal cancer

^*^Due to Adverse Events, PD and therapeutic procedures not permitted. **Patients not evaluated. Due to Adverse Events (*n* = 2), Death (*n* = 4), PD (*n* = 2), Investigator’s decision (*n* = 1) and other reasons (*n* = 3)

### Hypertension analysis

**Fig. 2:**
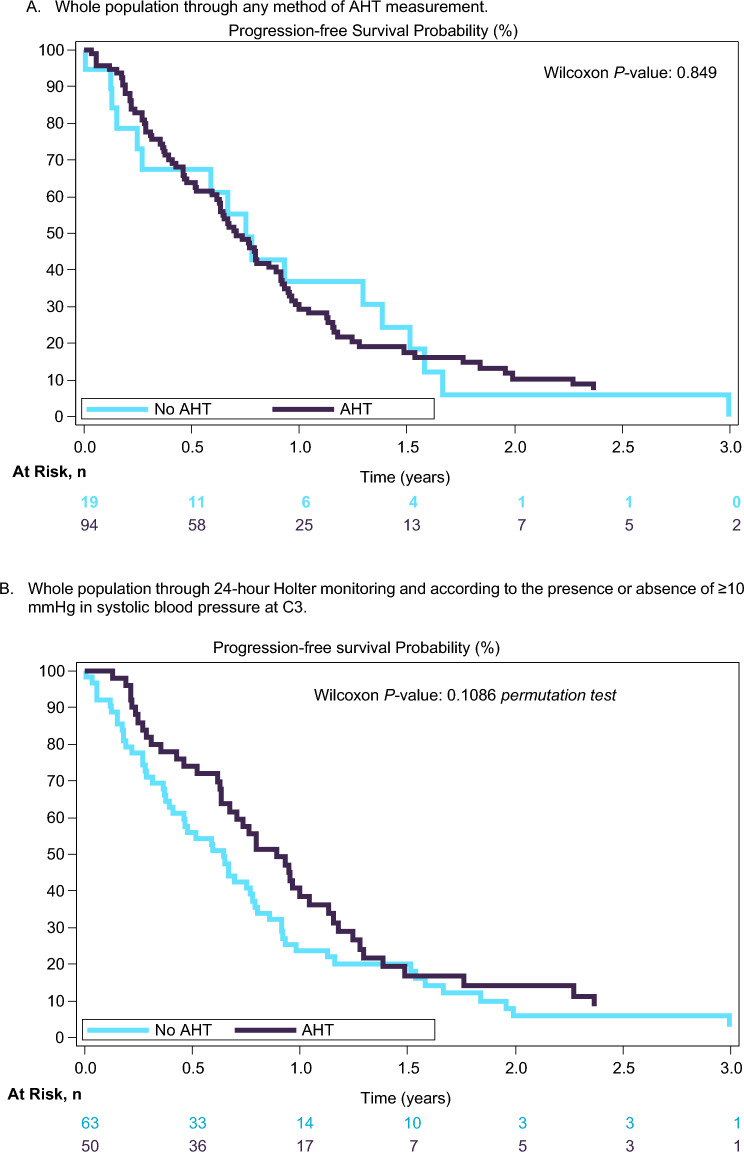

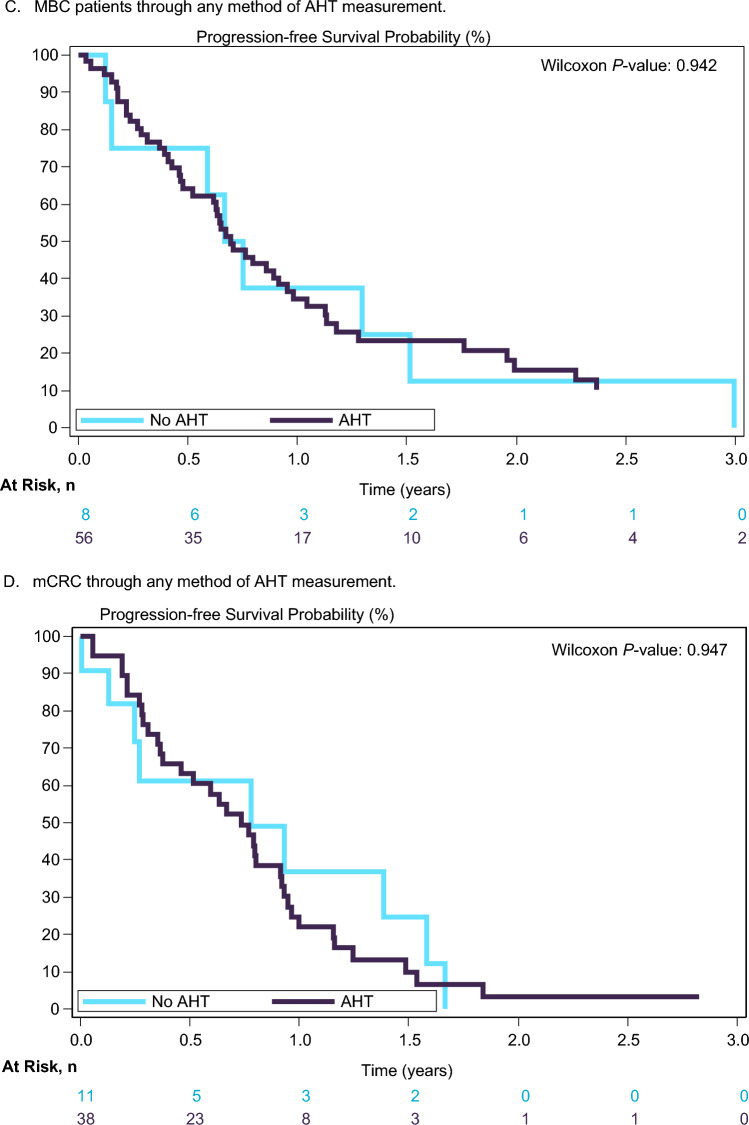
**A**–**D** Progression-free survival according to the presence or absence of high blood pressure levels. **A** Whole population through any method of AHT measurement. **B** Whole population through 24-h Holter monitoring and according to the presence or absence of ≥10 mmHg in systolic blood pressure at C3. **C** MBC patients through any method of AHT measurement. **D** mCRC through any method of AHT measurement

The AHT incidence according to the guidelines, was not a predictive factor of PFS in both MBC and mCRC patients treated with CT/BVZ. Figure 2 shows the K–M curves for PFS. There were also not statistically significant differences in tumor response according to presence or absence of AHT measured by any of the previously mentioned methods.

To assess the increase of BP levels, the differences in absolute value between BP measurements of C1–C3 and at baseline were reviewed considering several cut–off points. Using a cut-point model, we observed that the variation of ≥10 mmHg in SBP (24-h Holter) at C3 compared to baseline was not statistically significantly (Wilcoxon *P*-value = 0.1086 by permutation test) associated with PFS (Fig. 2B). When considering a difference in SBP (24-h Holter) of ≥10 mmHg after the start of CT/BVZ, using clinical variables for adjusting (tumor type, gender, previous AHT, age and prior therapy for AHT), a multivariate Cox proportional hazards regression model did not demonstrate a statistically significant difference for PFS (*P*-value = 0.1347); all variables included in the model were also found to be non-significant (*Supplementary Table 4*).

A cut-point model to correlate the same BP levels cut-off points with BOR was also performed (*n* = 98), and a statistically significant association was observed between BOR and the variation of ≥10 mmHg in SBP (24-h Holter) compared to baseline (Chi-square *P*-value = 0.0461 by permutation test).

### Safety

All G3 and G4 AEs related to CT/BVZ are detailed in *Supplementary Table 5*. Grade 3 AHT was reported in 11 (8%) patients, 9% and 7% in MBC and mCRC cohorts, respectively.

### Differential germline DNA methylation profiling in patients with secondary AHT

Methylation profiling was carried out in 32 patients, distributed in 4 experimental groups (8 patients/each) according to their AHT history and BP levels increase upon treatment (≥10 mmHg increase in SBP at any cycle vs. baseline).

We identified 27 sites with significantly different methylation status in pre-treatment samples in patients showing secondary AHT vs. those without increased BP levels after treatment. This difference was independent from AHT history (*Supplementary Fig. 1A–B*). Most of these sites (25 of 27) were hypermethylated in patients developing secondary AHT. The heatmap analysis suggested that sites showed a high intersite correlation degree (*Supplementary Fig. 1C*).

**Fig. 3:**
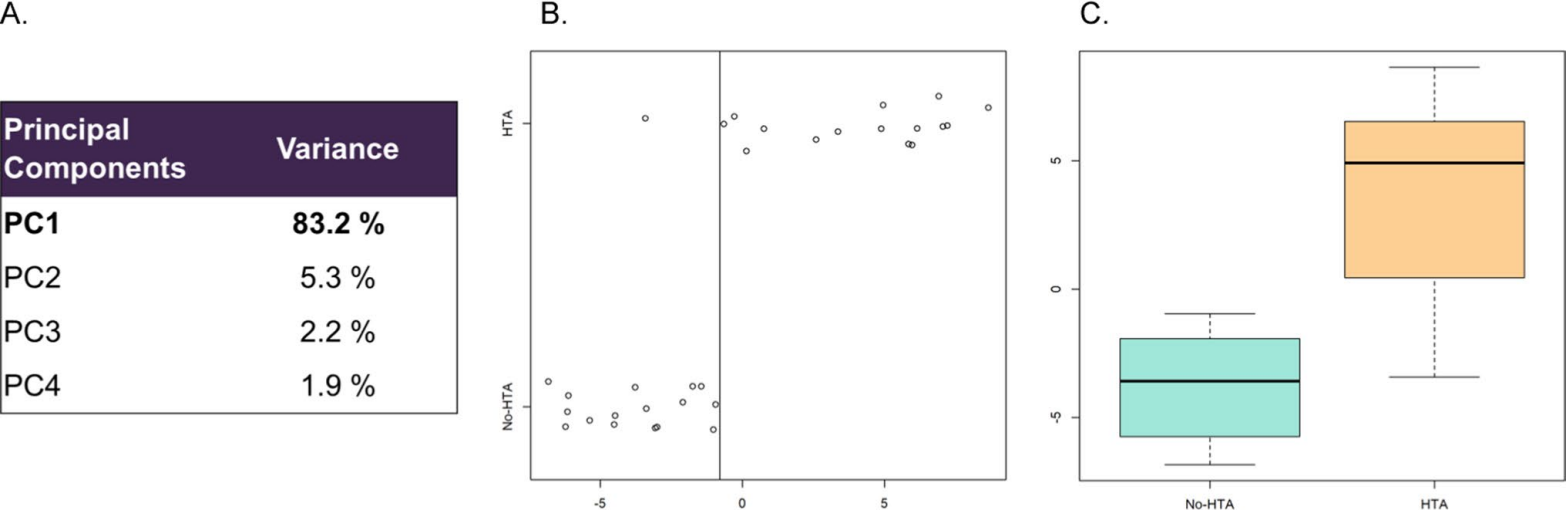
**A**–**C** Definition of a hypertension-predictive score based on principal components (PC) analysis. **A** PC1 explains 83% of the information provided by the variables. **B** Strip chart, and **C** boxplot distribution of the 32 patients according to PC1

We used PC analysis to generate a score to distinguish between patients with and without secondary AHT based on the methylation status of the 27 sites (Fig. 3). PC1 explains 83% of the information provided by the variables (Fig. 3A) and distribute patients into two categories (with/without secondary AHT) (Fig. 3B–C).

## Discussion

Hypertension is a common AE with BVZ, as well as with other antiangiogenic agents. However, various classifications for AHT grading, and different methods of BP levels measurement have been used in previous retrospective studies, preventing firm conclusions regarding the AHT incidence or its potential role as a surrogate efficacy marker for anti-VEGF agents [10].

Antiangiogenic drugs combined with CT, with BVZ as the major representative because of its pioneer role and profusion in publications, held the promise of increasing the efficacy in different tumors, including MBC and mCRC. The benefit of adding BVZ to CT has later proven to be lower than expected. This limited benefit, the lack of classical predictive factors for BVZ, and the emergence of dynamic predictive factors as potential methods of patients’ selection for targeted therapies, grounded the deeper evaluation of AHT incidence during treatment as a potential tool to optimize the use of BVZ in MBC and mCRC patients. Furthermore, retrospective analyses [11] as well as meta-analyses [12] suggested AHT as a potential biomarker for efficacy of BVZ-containing treatment, but prospective studies with formal regular BP levels measurements to confirm this hypothesis were lacking.

In this prospective observational study including MBC and mCRC patients receiving CT/BVZ, the AHT incidence, defined according to guidelines, was neither a predictive factor for PFS nor for BOR. However, the study suggested that the increase in SBP ≥ 10 mmHg, recorded with 24-h Holter device, may be a predictive factor for BOR. Germline DNA methylation could also be a potential surrogate predictor of BVZ efficacy.

Once we could not correlate the AHT, with PFS to CT/BVZ, we evaluated several cut-offs of differences in either SBP or DBP between baseline and at C3 measurements. We could not find any association, but we found out that the variation of ≥10 mmHg in SBP (24-h Holter) was statistically significantly associated with BOR. The translation of this finding to routine clinical practice as the method to select patients for BVZ-containing treatment seems unlikely. Performing 24-h Holter monitoring to all patients with BVZ administration, the narrow margin of the difference in BP levels considered relevant (10 mmHg) in a secondary analysis once we confirmed that the primary endpoint was no met, the limited sample size in our study, the dynamic nature of the predictive factor, and the fact that BVZ is mostly combined with other antineoplastic agents when used against MBC and mCRC, may all well be considered enough reasons to rule this option out in clinical practice.

We found that modifications of secondary AHT upon CT/BVZ were associated with changes in methylation patterns across the genome. We identified 27 DNA sites whose methylation status was significantly associated with secondary AHT during treatment. It is possible to define a predictive methylation profile for AHT secondary to BVZ-based treatment. Future studies should establish a correlation between this profile and the antiangiogenic treatment efficacy.

Antiangiogenic drugs combined with CT, with BVZ as major representative, held the promise of increasing the efficacy in different tumor types. Our study is unique, including two tumor types where BVZ was combined with CT, with regular measurement of BP levels with several methods, aiming to confirm the association between AHT and CT/BVZ efficacy. Nevertheless, we must accept some limitations in our investigation. Statistical assumption, with an expected high difference in PFS depending on the event of AHT, the fact that two different solid tumors were included, should be considered as main limitations. Further evaluation of the impact of specific treatment on AHT as well as the consideration of grading AHT could have improved its quality. The fact that BVZ was used combined with CT in two different indications may introduce some confounding factors. Even in a clinical study scenario, 30 of 143 patients (21%) were excluded of the efficacy analysis due to several reasons, 53% because of incomplete BP levels evaluation as per study protocol requirements, pointing out the difficulty of performing this sort of rigorous BP levels measurements in routine clinical practice. We needed to search differences in BP levels measurements with several cut-offs to finally find a ≥10 mmHg variation in SBP (24-h Holter monitoring) with respect to baseline as statistically significantly associated with an improvement in BOR after CT/BVZ.

BVZ-induced vascular changes are thought to confer survival benefit in responding patients, and these changes are thought to cause AHT in many patients [13] Since BVZ causes a reduction in both vascular density and nitric oxide (NO) production) [14], these are suspected to be causal factors in the pathogenesis of AHT following treatment [15–17].

Like our findings, the presence of AHT has been reported to be a positive prognostic biomarker for survival improvement in patients receiving antiangiogenic therapy. However, there is a need to elucidate practical issues related to the timing and value of BP levels that best predicts survival. Previous studies have shown that significant AHT and early onset of AHT, within 2 [17], 4 [18] or 6 weeks of treatment initiation [19], may be associated with improved survival. These studies do not detail specific BP monitoring and it is unclear whether patients who do not develop significant AHT in the initial phase require alterations in the medication regimen [18].

Of the 850,000 methylation sites analyzed, distributed throughout the genome, 27 were identified (18 of them corresponding to the genes FMNL2, METTL3, ACOT6, SCARNA20, PREX1, DNAI2, RAET1G, KCNJ8, GDF7, SYNPO2, CUGBP1, FRMD8, MKL2, HIF1A, TMEM177, UTP23, PXK and TNPO1) that were associated with the development of early tumor shrinkage secondary to BVZ. Among the genes identified are some linked to BP regulation and angiogenesis and AHT such as HIF1A or METTL3 [20, 21]. Future studies should clarify the predictive role to antiangiogenic treatments of this methylation profile of 27 genes related to AHT secondary to BVZ-containing treatment.

## Conclusion

In conclusion, this prospective observational study designed to evaluate AHT as a potential predictive factor for the efficacy of CT/BVZ in terms of PFS and BOR, in MBC and mCRC patients in the first-line scenario, failed to confirm this hypothesis; however, an increase of ≥10 mmHg in SBP level measured with a 24-h Holter device was associated to BOR. The translation of this finding to routine clinical practice as the method to select patients for BVZ-containing treatment seems unlikely.

## Supplementary Information

Below is the link to the electronic supplementary material.

Supplementary file1 (DOCX 574 KB)

## Data Availability

The datasets used and/or analyzed during the current study are available from the corresponding author on reasonable request.
