# Correction: Hypertension as predictive factor for bevacizumab-containing first-line therapy in metastatic breast and colorectal cancer in BRECOL (GEICAM/2011-04) study

**DOI:** 10.1007/s12094-025-04113-7

**Published:** 2025-11-25

**Authors:** Álvaro Rodríguez-Lescure, Javier Gallego, Pilar Garcia-Alfonso, Bartomeu Massuti, Raúl Márquez, Lourdes Calvo, Pedro Sánchez-Rovira, Antonio Antón, José Ignacio Chacón, Eva Ciruelos, Jose Juan Ponce, Ana Santaballa, Manuel Valladares-Ayerbes, María Rosario Dueñas, Vicente Alonso, Jorge Aparicio, Sara Encinas, Luis Robles, María José Escudero, Rosalía Caballero, Susana Bezares, María Victoria García-Ortiz, Teresa Morales-Ruiz, Juan de la Haba-Rodriguez

**Affiliations:** 1 Medical Oncology Department, Hospital General Universitario de Elche, Carrer Almazara, 11, 03203 Elche, Alicante Spain; 2 GEICAM Spanish Breast Cancer Group, Madrid, Spain; 3 Instituto de Investigación Sanitaria Gregorio Marañón, Facultad de Medicina de la Universidad Complutense de Madrid, Hospital General Universitario Gregorio Marañón, Madrid, Spain; 4 Instituto de Investigación Sanitaria y Biomédica de Alicante (ISABIAL), Hospital General Universitario Dr. Balmis, Alicante, Spain; 5 MD Anderson Cancer Center Madrid, Madrid, Spain; 6 Complejo Hospitalario Universitario de A Coruña, A Coruña, Spain; 7 Hospital Universitario de Jaén, Jaén, Spain; 8 Instituto de Investigación Sanitaria de Aragón (IISA), Hospital Universitario Miguel Servet, Universidad de Zaragoza, Saragossa, Spain; 9 Hospital Universitario de Toledo, Toledo, Spain; 10 Hospital Universitario, 12 de Octubre, Madrid, Spain; 11 Hospital Universitario y Politécnico La Fe, Valencia, Spain; 12 Instituto de Biomedicina de Sevilla (IBiS), Hospital Universitario Virgen del Rocío, Seville, Spain; 13 Instituto Maimónides de Investigación Biomédica (IMIBIC), Hospital Universitario Reina Sofía, Universidad de Córdoba, Córdoba, Spain; 14 Oncology Biomedical Research National Network (CIBERONC-ISCIII), Madrid, Spain; 15 Maimónides Biomedical Research Institute of Córdoba (IMIBIC), Córdoba, Spain; 16 Medical Oncology Department, Reina Sofía University Hospital, Córdoba, Spain; 17 Cancer Network Biomedical Research Center (CIBERONC), Madrid, Spain; 18 Department of Genetics, University of Córdoba, Córdoba, Spain; 19 Reina Sofía University Hospital, Córdoba, Spain

**Correction: Clinical and Translational Oncology (2024) 26:1896–1907** 10.1007/s12094-024-03411-w

In this article María Victoria García-Ortiz at affiliations ‘Maimónides Biomedical Research Institute of Córdoba (IMIBIC), Córdoba, Spain; Medical Oncology Department, Reina Sofía University Hospital, Córdoba, Spain; Cancer Network Biomedical Research Center (CIBERONC), Madrid, Spain’ was missing from the author list.

Teresa Morales-Ruiz at affiliations ‘Department of Genetics, University of Córdoba, Córdoba, Spain; Maimónides Biomedical Research Institute of Córdoba, (IMIBIC), Córdoba, Spain; Reina Sofía University Hospital, Córdoba, Spain’ was missing from the author list.

In this article the ‘Authors’ contributions’ statement was incorrectly given as ‘ARL, JG and JdH: Conception and design, provision of study material or patients, collection and/or assembly of data, financial support, data analysis and interpretation, manuscript writing and final approval of manuscript. PGA, BM, RM, LC, PSR, AA, JIC, EC, JP, AS, MV, MRD, VA, JA, SE, LL and LR: provision of study material or patients, collection and/or assembly of data and final approval of manuscript. MJE, RC and SB: data analysis and interpretation, manuscript writing and final approval of manuscript’ but should have been ‘ARL, JG and JdH: Conception and design, provision of study material or patients, collection and/or assembly of data, financial support, data analysis and interpretation, manuscript writing and final approval of manuscript. PGA, BM, RM, LC, PSR, AA, JIC, EC, JP, AS, MV, MRD, VA, JA, SE, LR, MVG and TM: provision of study material or patients, collection and/or assembly of data and final approval of manuscript. MJE, RC and SB: data analysis and interpretation, manuscript writing and final approval of manuscript.’

The original article has been corrected.

