# Supplementary material for: Hypertension as predictive factor for bevacizumab-containing first-line therapy in metastatic breast and colorectal cancer in BRECOL (GEICAM/2011-04) study
Source: Clin Transl Oncol. 2024 Apr 5;26(8):1896–907. doi: 10.1007/s12094-024-03411-w (PMC11249439; doi:10.1007/s12094-024-03411-w)
Supplement: Supplementary file 1 — Supplementary file1 (DOCX 574 KB) [file 12094_2024_3411_MOESM1_ESM.docx]

#### SUPLEMENTARY MATERIAL

*Supplementary Methods*

*Germline DNA methylation analysis:*

Germline DNA was extracted from pre-treatment peripheral blood samples and its global methylation status was checked by agarose gel upon MspI/HpaII digestion(1). Germline DNA (500 ng) was treated with sodium bisulfite to convert cytosine to uracil and then hybridized to the Infinium MethylationEPIC BeadChip (Illumina®) according to manufacturer’s protocol. Raw intensity data files (idat files) were processed using the R package ChAMP version 2.9.10(2). Probes were filtered(3) and intracell type normalization was done using beta-mixture quantile (BMIQ) method(4).

*Supplementary Table 1. Criteria for arterial hypertension diagnosis.*

|  | SBP (mmHg) |  | DBP (mmHg) |
| --- | --- | --- | --- |
| At hospital office | >140 | and/or | >90 |
| 24-hour Holter device monitoring | >130 |  | >80 |
| 24-hour Holter device daytime monitoring | >135 |  | >85 |
| 24-hour Holter device night-time monitoring | >120 |  | >70 |
| At home, a pharmacy, or a primary healthcare center | >135 |  | >85 |

Abbreviations: SBP = Systolic Blood Pressure. DBP = Diastolic Blood Pressure. mmHg = millimeters of Mercury.

#### Supplementary Table 2. Reasons for ending the study treatment.

|  | MBC (n=64) | mCRC (n=49) | Total (n=113) |
| --- | --- | --- | --- |
| n | 62 | 49 | 111 |
| Missing, n | 2 | 0 | 2 |
| AE, n (%)   - Proteinuria (n=3) - Renal failure of G3 (n=1) - DVT (n=1) - TEE of G4 (n=1) - Atrial fibrillation of G2 (n=1) - Neuropathy of G4 (n=1) - Ictus (n=1) - Confusion (n=1) - Perianal fistula (n=1) - Mandibular fistula (n=1) - Foot ulcer (n=1) - Listeria infection (n=1) - Clinical worsening (n=1) - Neuropathy and dystrophy (n=1) - SAE (n=1) | 11 (18) | 6 (12) | 17 (15) |
| Death, n (%) | 3 (5)^a^ | 1 (2)^b^ | 4 (4) |
| Further treatment refused, n (%) | 0 (0) | 1 (2) | 1 (1) |
| Investigator decision, n (%) | 4 (7) | 8 (16) | 12 (11) |
| Lost to follow-up, n (%) | 1 (2) | 0 (0) | 1 (1) |
| Other reason, n (%) | 2 (3) | 10 (20) | 12 (11) |
| PD, n (%) | 41 (66) | 22 (45) | 63 (57) |
| Therapeutic procedure not permitted, n (%) | 0 (0) | 1 (2) | 1 (1) |

Abbreviations: MBC = metastatic breast cancer. mCRC = metastatic colorectal cancer. n = number of patients. AE = adverse event. G = Grade. DVT = deep venous thrombosis. TEE = thromboembolic event. SAE = serious adverse event. PD = progressive disease.

#### Supplementary Table 3. Dose modifications of the study treatment (omissions, delays, and reductions).

| Dose modifications for chemotherapy regimens | | | |
| --- | --- | --- | --- |
|  | MBC (n=64) | mCRC (n=49) | Total (n=113) |
| Dose modification, n (%) | | | |
| No | 49 (77) | 23 (47) | 72 (64) |
| Yes | 15 (23) | 26 (53) | 41 (36) |
| Dose reduction, n (%) | | | |
| No | 59 (92) | 37 (76) | 96 (85) |
| Yes | 5 (8) | 12 (24) | 17 (15) |
| Dose delay, n (%) | | | |
| No | 54 (84) | 30 (61) | 84 (74) |
| Yes | 10 (16) | 19 (39) | 29 (26) |
| Dose omission, n (%) | | | |
| No | 56 (87) | 45 (92) | 101 (89) |
| Yes | 8 (13) | 4 (8) | 12 (11) |
| Reason of modifications, n (%) | | | |
| AE | 11 (17) | 21 (43) | 32 (28) |
| Investigator’s decision | 4 (6) | 1 (2) | 5 (4) |
| Administrative reason | 1 (2) | 2 (4) | 3 (3) |
| Patient’s decision/Vacation | 1 (2) | 1 (2) | 2 (2) |
| Radiotherapy | 0 (0) | 1 (2) | 1 (1) |
| Not specified | 2 (3) | 3 (6) | 5 (4) |
| Dose modifications for bevacizumab administration | | | |
|  | MBC (n=64) | mCRC (n=49) | Total (n=113) |
| Dose modification, n (%) | | | |
| No | 50 (78) | 29 (59) | 79 (70) |
| Yes | 14 (22) | 20 (41) | 34 (30) |
| Dose reduction, n (%) | | | |
| No | 61 (95) | 49 (100) | 110 (97) |
| Yes | 3 (5) | 0 (0) | 3 (3) |
| Dose delay, n (%) | | | |
| No | 53 (83) | 31 (63) | 84 (74) |
| Yes | 11 (17) | 18 (37) | 29 (26) |
| Dose omission, n (%) | | | |
| No | 59 (92) | 46 (94) | 105 (93) |
| Yes | 5 (8) | 3 (6) | 8 (7) |
| Reason of modifications, n (%) | | | |
| AE | 9 (14) | 16 (33) | 25 (22) |
| Investigator’s decision | 3 (5) | 0 (0) | 3 (3) |
| Administrative reason | 3 (5) | 2 (4) | 5 (4) |
| Patient’s decision/Vacation | 1 (2) | 1 (2) | 2 (2) |
| Radiotherapy | 0 (0) | 1 (2) | 1 (1) |
| Weight loss | 1 (2) | 0 (0) | 1 (1) |
| Surgical procedure | 0 (0) | 1 (2) | 1 (1) |
| Not specified | 1 (2) | 1 (2) | 2 (2) |

Abbreviations: MBC = metastatic breast cancer. mCRC = metastatic colorectal cancer. n = number of patients. AE = adverse event.

# *Supplementary Table 4.* *Multivariate Cox proportional hazards regression model analysis for Progression-free Survival (PFS). Saturated model.*

|  | PFS | |
| --- | --- | --- |
| Variable | Hazard Ratio [95% CI] | P value |
| >10mmHg of difference in SBP (24-hour Holter) (ref: Yes) | 0.73 [0.48, 1.10] | 0.14 |
| Disease (ref: MBC) | 1.02 [0.57, 1.80] | 0.96 |
| Gender (ref: Male) | 1.16 [0.64, 2.13] | 0.62 |
| Previous AHT (ref: No) | 1.10 [0.61, 2.01] | 0.75 |
| Age (years) | 1.01 [0.99, 1.03] | 0.26 |
| Prior treatment for AHT (ref: No) | 1.34 [0.74, 2.45] | 0.34 |

CI denotes confidence interval, SBP systolic blood pressure, MBC metastatic breast cancer and, AHT arterial hypertension.

#### Supplementary Table 5. Grade 3 and 4 adverse events related to the treatment in the safety population (≥5% incidence).

|  | MBC (n = 75)  n (%) | | mCRC (n = 60)  n (%) | | Total (n = 135)  n (%) | |
| --- | --- | --- | --- | --- | --- | --- |
| Grade of severity  (NCI-CTCAE version 4.0) | G3 | G4 | G3 | G4 | G3 | G4 |
| Any AE | 23 (31) | - | 17 (28) | 6 (10) | 40 (30) | 6 (4) |
| GGT increased | 7 (9) | - | 1 (2) | - | 8 (6) | - |
| Neutropenia | 4 (5) | - | 8 (13) | 3 (5) | 12 (9) | 3 (2) |
| Arterial hypertension | 7 (9) | - | 4 (7) | - | 11 (8) | - |

Thirty-five patients experienced at least one serious AE (SAE) and, in 14 patients, these events were related to CT/BVZ. Except for diarrhea, the rest of SAEs were reported only in one patient (<1% each).

Abbreviations: MBC = metastatic breast cancer. mCRC = metastatic colorectal cancer. G = grade. AE = adverse event. GGT = gamma glutamyl transpeptidase.

*Supplementary Figure 1 (A-D). Germline DNA Methylation profiling in pretreatment blood samples according to the development of secondary hypertension.*

Methylation profiling was carried in 4 experimental groups (8 patients/each, n=32) according to their AHT history and BP levels increase upon treatment (≥10mmHg increase in SBP at any cycle vs. baseline): group 1 (AHT history/BP increase), group 2 (no AHT history/BP increase), group 3 (AHT history/no BP increase), and group 4 (no AHT history/no BP increase). A) Identification of 27 sites with significantly different methylation status between patients showing vs. not showing secondary hypertension independently of prior hypertension history. B) Boxplot distribution of the methylation status of 4 sites (out of the 27 identified) between the 4 experimental groups. C) Heatmap of the methylation status of the 27 identified sites in the 32 patients according to their hypertension status (y/n) shows a high intersite correlation degree. D) Genomic localization of identified sites: nine methylation sites were mapped in intergenic regions whereas 18 in known protein-coding genes, 9 of them in regulatory regions and 9 in the gene body.

A.

B.


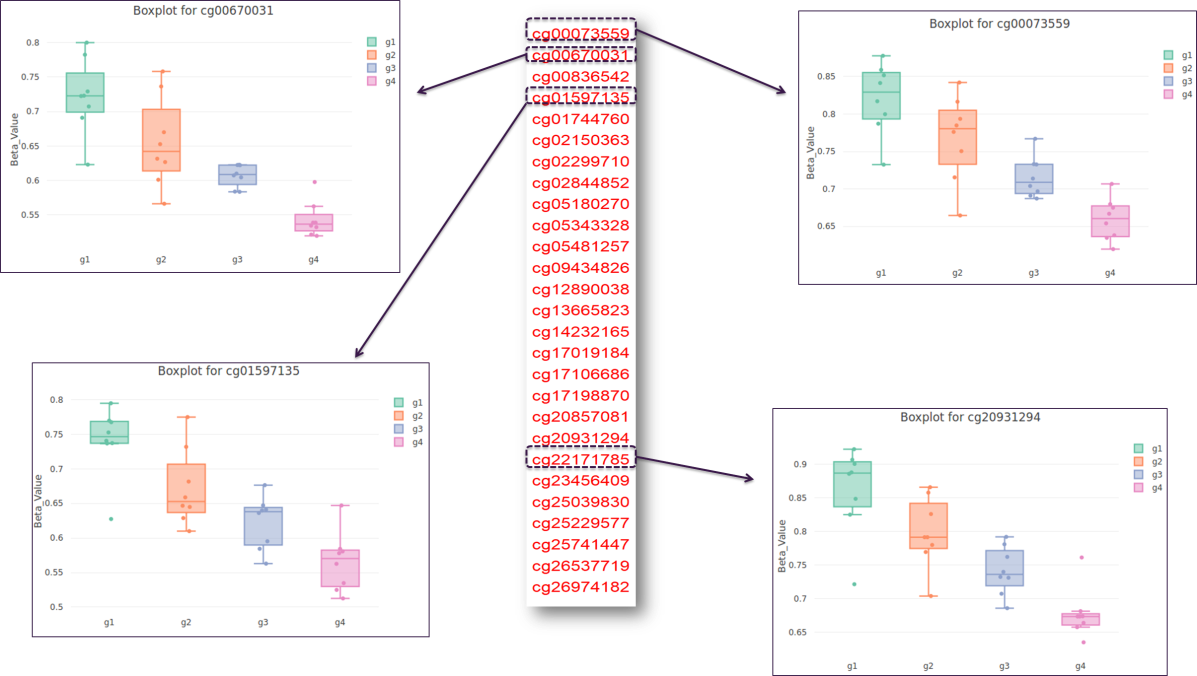


C.


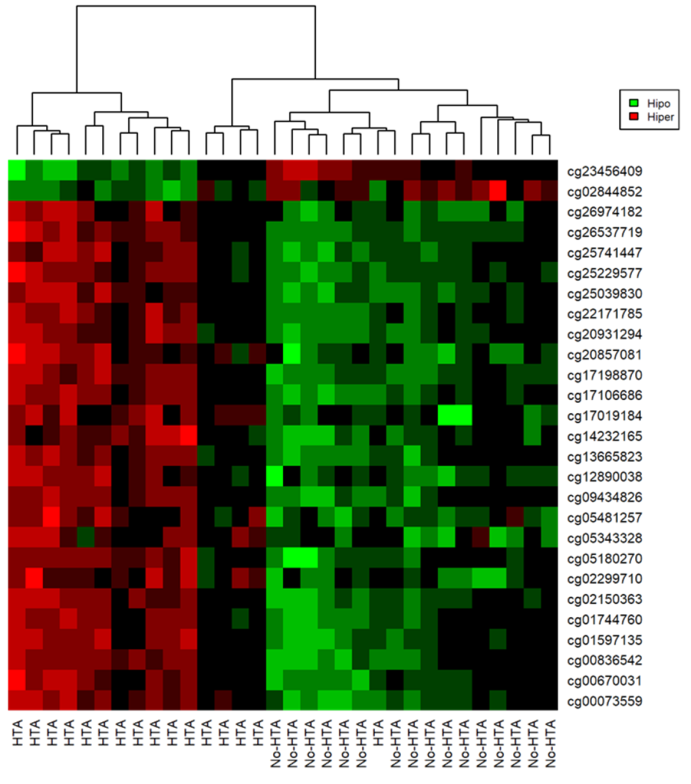


D.


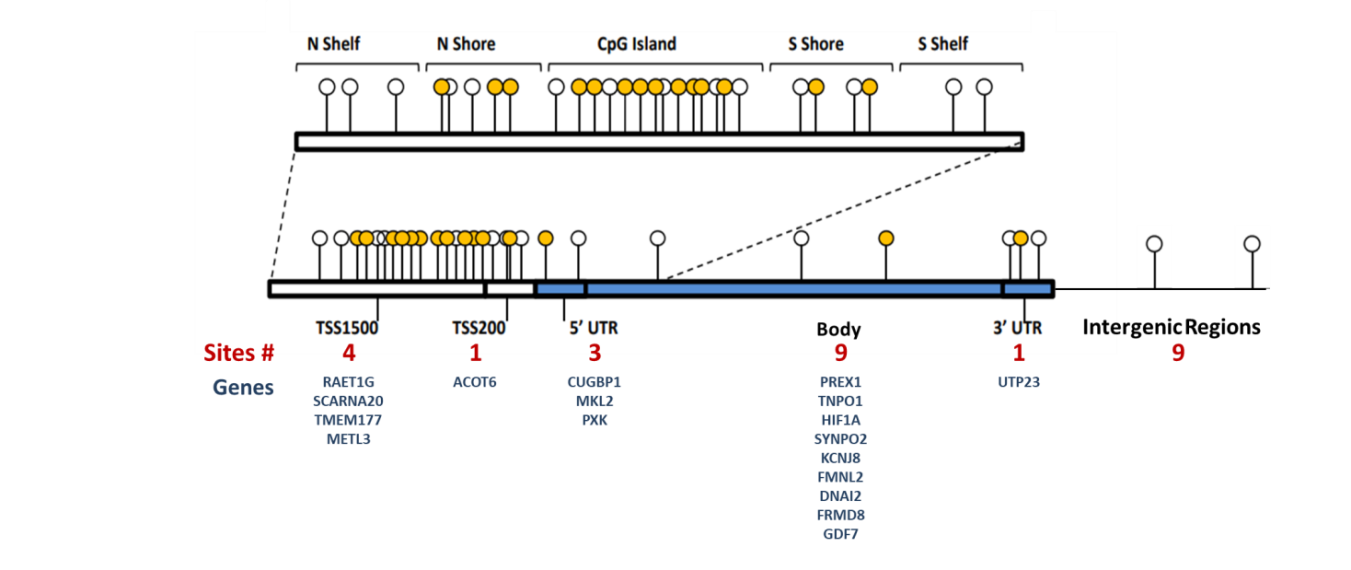


**REFERENCES**

1. Shiratori H, Feinweber C, Knothe C, Lotsch J, Thomas D, Geisslinger G, et al. High-Throughput Analysis of Global DNA Methylation Using Methyl-Sensitive Digestion. PLoS One. 2016;11(10):e0163184.

2. Morris TJ, Butcher LM, Feber A, Teschendorff AE, Chakravarthy AR, Wojdacz TK, Beck S. ChAMP: 450k Chip Analysis Methylation Pipeline. Bioinformatics. 2014;30(3):428-30.

3. Zhou W, Laird PW, Shen H. Comprehensive characterization, annotation and innovative use of Infinium DNA methylation BeadChip probes. Nucleic Acids Res. 2017;45(4):e22.

4. Teschendorff AE, Marabita F, Lechner M, Bartlett T, Tegner J, Gomez-Cabrero D, Beck S. A beta-mixture quantile normalization method for correcting probe design bias in Illumina Infinium 450 k DNA methylation data. Bioinformatics. 2013;29(2):189-96.
